# Exercise Rehabilitative Approach to Functional Improvement in Adult Idiopathic Scoliosis: A Functional Movement Screen-based Program

**Published:** 2018-07

**Authors:** Kewwan KIM, David MULLINEAUX, Kyoungkyu JEON

**Affiliations:** 1. Division of Sport Science, College of Arts and Physical Education, Incheon National University, Incheon, Republic of Korea; 2. School of Sport and Exercise Science, University of Lincoln, Lincoln, United Kingdom; 3. Sport Science Institute, Incheon National University, Incheon, Republic of Korea

## Dear Editor-in-Chief

Scoliosis is an idiopathic disease characterized by lateral deviation or rotation of the spine, and it is defined as an abnormal lateral curvature of the spine, including spinal rotation and thoracic kyphosis (1, 2). It usually develops idiopathically in adolescence, but inadequate management throughout one’s growth makes the condition chronic due to lumbar instability and degenerative changes, resulting in functional instability (3). Such structural and functional problems cause physical deformity, and as a result, these problems become coupled with physical limitation, reduced physical stability, and musculoskeletal pain (4, 5). Thus, therapeutic exercise programs are required to enhance the functional capacity to increase internal and external stability of the spine and to prevent scoliosis. Therefore, this study aimed to provide scientific contributions for functional improvement in scoliosis by examining the effects of a functional movement screen (FMS)-based exercise program on the improvement of abnormal lateral curvature and pelvic asymmetry of the spine in patients with adult idiopathic scoliosis (AIS).

Twenty-five adult male patients with AIS willing to participate in a 10-week exercise program to reduce asymmetrical curvature and pain were enrolled in this study in 2014.

All participants provided written informed consent, and the Institutional Review Boards at Incheon National University approved the study.

To analyze abnormal spinal curvature and pelvic asymmetry in AIS, trunk inclination, torsion, and rotation; pelvic inclination, torsion, rotation, and symmetry; and the scoliosis angle in the sagittal and coronal planes were assessed using three-dimensional spinal structural analysis (Formetric 4D, DIERS International GmbH, Schlangerbad, Germany). Obtaining this information using equipment enables accurate and quick measurements because four posterior anatomical points (i.e., the vertebra prominent, left and right posterior superior iliac spine (PSIS), and middle dimple of the PSIS) are imaged through surface topography using rasterstereography. Furthermore, when compared with radiography, rasterstereography produces highly reproducible and objective data without the risk of radiation exposure (6), making it a useful method for diagnosing spinal asymmetry and scoliosis.

The FMS (Functional Movement Screen System Inc., Chatham, VA, USA) was used for the exercise that was designed to recover stability and alleviate curvature in AIS. The FMS was used to assess seven movement pattern (deep squat, hurdle step, in-line lunge, shoulder mobility, straight-leg raise, trunk stability push-up, rotary stability) and 3 clearing tests (impingement, press-up, posterior rocking). The 10-week program included active static training based on body weight and passive manual training for postural adjustment, with three 60-min sessions per week. Three-dimensional structural analysis was performed to evaluate changes of functional improvement in AIS after the 10-week FMS-based exercise. The mean (M) and standard deviation (SD) were calculated for every measurement category and paired t-test was used to analyze changes in every variable after the 10-week FMS program. All statistical analyses were performed using SPSS 23.0 (SPSS Inc., Chicago, IL, USA). A *P*-value <0.05 was considered statistically significant.

There were significant changes in the pelvic obliquity (*P*=0.005) and symmetry (*P*=0.000), and scoliosis angle (*P*=0.000) (Table 1). Although statistically significant, the FMS-based exercise was effective in improving other parameters, suggesting the FMS-based preservative exercise may be beneficial for enhancing abnormal curvature and asymmetry of the spine.

**Table 1:** Results of spinal curvature by three-dimensional spinal structural analysis

***Variables***		***Pre-test***	***Post-test***
Trunk	Inclination (deg)	1.85±2.51	1.80±1.78
	Imbalance (deg)	−0.27±1.11	−0.08±1.12
	Rotation (deg)	3.46±7.25	9.81±4.22
Pelvic	Obliquity (deg)	−2.23±3.92	−0.33±2.71^**^
	Torsion (deg)	2.55±3.06	1.65±1.55
	Rotation (deg)	−1.85±2.99	−0.86±2.87
	Symmetry (deg)	13.95±4.47	9.81±4.22^***^
Scoliosis Angle (deg)		13.48±2.99	9.44±3.50^***^

Values are Mean±SD,

** *P*<0.01,

*** *P*<0.001

Various active preservative exercise programs should be developed and based on scientific grounds, and these problems may be used to rehabilitate patients with lesions (e.g., lordosis and scoliosis). Furthermore, a three-dimensional method to an examination of the prevalence of idiopathic scoliosis during the growth period would bring about scientific contributions to advancing public health.
